# Resiliency of EEG-Based Brain Functional Networks

**DOI:** 10.1371/journal.pone.0135333

**Published:** 2015-08-21

**Authors:** Mahdi Jalili

**Affiliations:** School of Electrical and Computer Engineering, RMIT University, Melbourne, Australia; University of Michigan, UNITED STATES

## Abstract

Applying tools available in network science and graph theory to study brain networks has opened a new era in understanding brain mechanisms. Brain functional networks extracted from EEG time series have been frequently studied in health and diseases. In this manuscript, we studied failure resiliency of EEG-based brain functional networks. The network structures were extracted by analysing EEG time series obtained from 30 healthy subjects in resting state eyes-closed conditions. As the network structure was extracted, we measured a number of metrics related to their resiliency. In general, the brain networks showed worse resilient behaviour as compared to corresponding random networks with the same degree sequences. Brain networks had higher vulnerability than the random ones (P < 0.05), indicating that their global efficiency (i.e., communicability between the regions) is more affected by removing the important nodes. Furthermore, the breakdown happened as a result of cascaded failures in brain networks was severer (i.e., less nodes survived) as compared to randomized versions (P < 0.05). These results suggest that real EEG-based networks have not been evolved to possess optimal resiliency against failures.

## Introduction

Networked structures are abundant and many real-world systems can be modelled as networks with nodes representing the individual units and edges representing the relations between them. In recent years, with tremendous progress in computing tools and database systems, techniques developed in *Network Science* have been applied to many real-world systems [1–3]. Graph theory tools have been extensively applied to the signals recorded from the brain [4, 5]. The brain can be described as a networked structure at both micro and macro levels. In brain networks, nodes represent the defined brain regions and the edges correspond to anatomical/functional relations between these regions. Anatomical brain networks can be studied using diffusion tensor imaging (DTI) [6] while techniques such as functional magnetic resonance imaging (fMRI), electroencephalography (EEG) and magnetocephalography (MEG) can be used to discover functional brain networks [7–9]. Analysis of brain networks in health and disease has revealed that their structure might be disrupted in brain disorders such as epilepsy [10], Parkinson’s disease [11], schizophrenia [9, 12], Alzheimer’s disease [13–15], and psychogenic non-epileptic seizures [16].

Complex networks might undergo component failure and lose some of their nodes and/or edges [17]. Component failures are often divided into two categories: errors and attacks. Errors are random failures, while attacks are targeted failures of (often important) nodes/edges. Many biological networks have shown to be robust against random errors that might happen in their structure [17–19]; however, they might not be enough resilient against intentional attacks to their hub components. Indeed, scale-free networks are fragile against attacks [17, 20], and many real-world networks are scale-free, i.e., their degree distribution is power-law. Often, structural properties of networks such as their global efficiency and the size of the largest connected component are used to study failure tolerance of networks [17, 20–22]. Dynamical properties such as synchronizability and cooperation have also been studied in networks undergoing errors and attacks [23, 24]. Component failure might have a devastating outcome when it results in a cascade of failures in other components [25–27]. Random or intentional failures in some components of a network might cause other components to go beyond their capacity. As a result, some other components might also fail and this process can lead to a cascaded failure, which breaks the network down and prevents it from proper functioning [28].

In this manuscript, we studied failure tolerance of EEG-based brain functional networks. Our study, to the best of our knowledge, is the first one reporting failure tolerance of brain functional networks. The EEGs recorded from 30 healthy subjects were used for the analysis. The properties of the brain networks were compared with properly randomized networks with the same degree-sequence as the original networks. Such network randomization strategy has been previously suggested to discover the motifs in networks [29]. We used correlation analysis [30] in order to obtain the network connectivity matrices. Our analysis revealed that brain networks are less efficient (in terms of communicability between the nodes) as compared to the randomized versions. They are also less resilient than random networks; the brain networks have higher vulnerability than random ones. Furthermore, cascaded failures have more devastating outcome in the brain networks than random ones.

## Methods

### A. EEG recording

We used the EEGs of 30 healthy subjects (age 33.4 ± 10.8; 17 men; all right-handed) without known neurological or psychiatric illness or trauma and without substance abuse or dependence. These subjects have been previously used as healthy controls in our projects on studying EEG signs of schizophrenia, Alzheimer’s disease and non-epileptic seizures [31–33]. All participants in this study were fully informed about the study and gave written consent. All the procedures conformed to the Declaration of Helsinki (1964) by the World Medical Association concerning human experimentation and were approved by the local ethics committee of Lausanne University.

The 3–4 minutes of resting-state eyes-closed EEG data were collected in a semi-dark room with a low level of environmental noise while each subject was sitting in a comfortable chair. The resting state EEGs were recorded with the 128-channel Geodesic Sensor Net (EGI, USA) with all the electrode impedances kept under 30 kΩ. The recordings were made with vertex reference using a low-pass filter set to 100 Hz. The signals were digitized at a rate of 1000 samples/s with a 12-bit analog-to-digital converter. They were further filtered (FIR, band-pass of 1–70 Hz, notch at 50 Hz), re-referenced against the common average reference, and segmented into non-overlapping epochs using the NS3 software (EGI, USA). Artefacts in all channels were edited off-line: first, automatically, based on an absolute voltage threshold (100 μV) and on a transition threshold (50 μV), and then by thorough visual inspection, which allowed us to identify and reject epochs or channels with moderate muscle artifacts not reaching threshold values. We also excluded from further analysis the outer sensors due to low signal to noise ratio. Finally, 111 sensors were used for further computation.

The interpretation of surface common average reference EEG is limited because of contamination by volume conduction and reference electrode effects [34]. In this work, we used correlation analsys to construct connectivity matrices, and it has been shown that correlation coefficients are significantly infleunced by volume conduction [35]. These unwanted effects were minimized with the high-resolution Laplacian transformed EEG signals, which isolates source activity under each sensor [35, 36]. To this end, at each sample, a 2-D spline was fitted to common-average-reference EEG, along the surface of the best-fit sphere [37]. For computing Laplacian transform of EEG signals, we used the CSD toolbox (psychophysiology.cpmc.columbia.edu/Software/CSDtoolbox). To obtain greater confidence in the correlation estimates, signals were segmented into non-overlapping 1-second epochs. The EEG time series were analyzed in conventional frequency bands including theta (3–7 Hz), alpha (7–13 Hz), beta (13–30 Hz) and gamma (30–50 Hz). We used a fifth order Elliptic bandpass filter with 0.5 dB of ripple in the passband and 20 dB of attenuation in the stopband.

### B. Constructing brain functional networks

The filtered EEG time series were used to obtain the connectivity structure of the functional brain networks. The first step in extracting the network structure from time series is to obtain the correlation (or dependency) matrix between the nodes, i.e., EEG sensor locations. We applied Pearson product momentum correlation coefficient for all electrode pairs, resulting in a 111 × 111 weighted correlation matrix for each subject. The correlation coefficient between sensors *i* and *j* can be obtained as

$$r_{ij}=\frac{\mathrm{cov}(i,j)}{\sqrt{\mathrm{var}(i)\mathrm{var}(j)}},$$ (1)

where cov(*i*,*j*) is the covariance between nodes *i* and *j*, and var(*i*) is the variance of node *i*. By averaging the absolute value of the correlation matrices over the artefact-free epochs, we computed a weighted correlation matrix for each subject.

The next step is to construct the functional brain networks based on the correlation matrices. Often, binary brain networks (with entries as 1 or 0) are studied [38]. A common method for constructing binary networks is to threshold the weighted correlation matrices: if the correlation between two nodes is larger than a certain threshold, the corresponding entry in the binary adjacency matrix is set to 1, otherwise to 0. Networks can be defined arbitrarily on the basis of different thresholds, but may not be comparable. Indeed, binarizing two correlation matrices based on a specific threshold value might result in two networks with different density values, i.e., number of links. An alternative approach is to build binary networks based on network density, that is, to threshold them in such a way that they have equal density values [15, 39]. We binarized the weighted correlation matrices (under different threshold values) such that they have the same number of links, i.e., with the same density. To this end, the density values varied from 0.02 to 0.3 with 0.01 steps.

### C. Resiliency metrics

**Fig 1:**
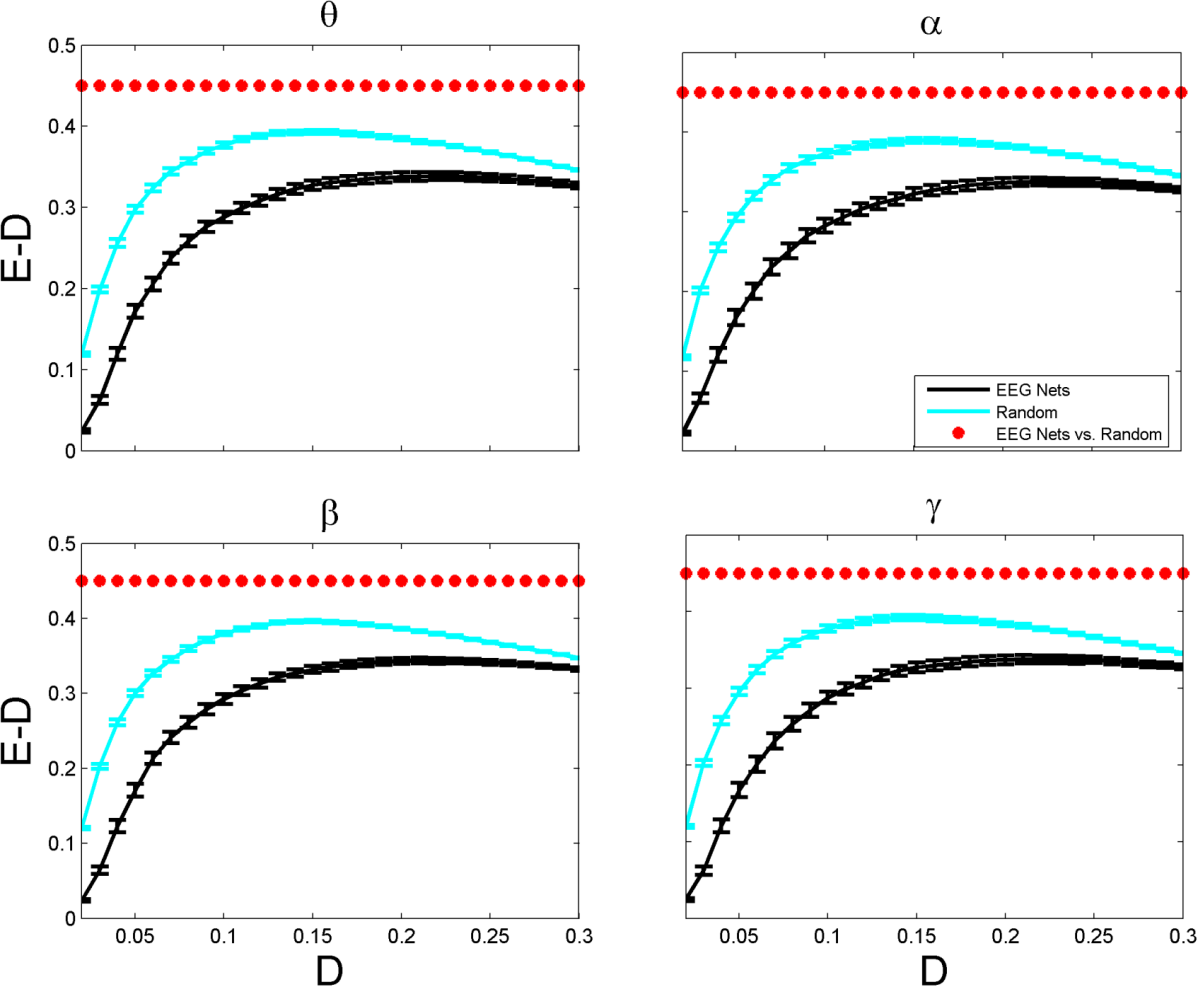
Efficiency-cost plots (Global Efficiency (*E*) minus Network Density (*D*)) of EEG-based brain networks as a function of density values. Mean values with bars corresponding to the standard errors of the efficiency-cost curves are plotted for different frequency bands including theta (3–7 Hz), alpha (7–13 Hz), beta (13–30 Hz) and gamma (30–50 Hz).The graphs show the metrics for the functional networks obtained from EEG time series of 30 healthy subjects (black lines) and the randomized networks (cyan lines). The randomized networks have the same degree sequence as the original networks. The red asterisk above the plots represent the density value for which the two populations have significantly different median (Wilcoxon’s ranksum test; P < 0.05).

Error and attack tolerance of complex networks is one of the major issues studied in network science [17, 21, 40]. This is often studied by observing how various network properties change as their components are removed randomly or intentionally. Among the properties studies in this context are the global efficiency and the size of the largest connected component. Global efficiency of a network is closely related to its integration properties [41]. Network integration is the ability of a network to combine the information of various parts. The global efficiency is defined as

$$E=\frac{1}{N(N-1)}\sum_{i,j}\frac{1}{l_{i,j}},$$ (2)

where *N* is the size of the network (*N* = 111 here) and *l*_*i*,*j*_ is the length of the shortest path between nodes *i* and *j*. Global efficiency is analogous to the average path length; it can be applied on both connected and disconnected networks, while the average path length cannot be computed for disconnected networks. It scales from 0 to 1; complete networks have global efficiency of 1, and very sparse networks with long paths between their nodes have global efficiency near 0. The density-efficiency analysis of networks can give useful information on their cost-economic structure. Both these metrics scale between 0 and 1, and substituting density from efficiency, as shown in [39], results in a peak-shaped curve (see Fig 1), which shows the density value for which the network has cost-efficient behavior. Indeed, as the network density increases (i.e., the number of edges increases), its efficiency also increase. However, by increasing the density from a certain value, the rate of increase in the efficiency decrease, and thus density-efficiency curve (efficiency minus density) declines. The density value at which the efficiency-cost curve reaches its maximum is often interpreted as the optimal density in terms of the economic wirings of the network.

Networks may undergo random and/or intentional failures in their components, and their resiliency against such a failure is of high importance for their proper functioning. If the performance of the network is associated to its efficiency, the vulnerability of a node would be the amount of drop in the performance when the node is removed from the network. More precisely, vulnerability of node *i* is calculated as

$$V_{i}=\frac{E-E_{i}}{E},$$ (3)

where *E* is the efficiency of the original network and *E*_*i*_ is the efficiency of the network after removal of node *i*. A measure for the network vulnerability is the maximum vulnerability for all its nodes

$$V=\max_{i}V_{i}\;;\;i=1,2,\ldots ,N.$$ (4)

Another frequently considered framework to study resiliency of complex networks is cascaded failures [26–28]. We considered a redistribution rule for the cascaded failures [26–28, 42, 43]. First, each node is associated with a capacity value. Here, we considered the capacity of each node to be a function of its load (betweenness centrality) in the original network. Node-betweenness centrality *B*_*i*_ is a centrality measure of node *i* in a graph, which shows the number of shortest paths making use of node *i* (except those between the *i*-th node with the other nodes) [44]. More precisely,

$$B_{i}=\sum_{p\neq i\neq q}\left(\Gamma_{pq}\left(i\right)/\Gamma_{pq}\right),$$ (5)

where Γ_*pq*_ is the number of shortest paths between nodes *p* and *q* and Γ_*pq*_(*i*) is the number of these shortest paths making use of the node *i*. Capacity of each node is calculated as

$$C_{i}=\left(1+c\right)B_{i},$$ (6)

where *c* > 0 is a control parameter determining the relation between the capacity and the initial load. The nodes become better tolerant (i.e., higher capacity) for higher values of *c*.

The algorithm used for cascaded failures is as follows. First, the betweenness centrality values were calculated, and the capacities were obtained for a chosen value for *c*. Then, the node with the highest betweenness value was removed from the network. Note that when a node is removed from the network, all its connecting edges are also removed. Then, the betweenness values were recalculated, and the nodes whose betweenness was higher than their capacity, were removed from the network. This process continued until no further removal was needed and a steady state solution was attained. Finally, the percentage of survived nodes (*S*) was calculated as an indicator of cascade depth; the higher the *S*, the better resilient the network against cascaded failures.

### D. Statistical assessments and correlation analysis

The attack tolerance of the brain functional networks was compared with that of properly randomized version of the networks. For each binary network, we randomized it by shuffling the edges such that the original nodes’ degree was kept in the randomized version. This randomization process was performed 100 times and the average values were used for statistical test. Parameters of EEG-based brain networks and their randomized versions were passed through Wilcoxon’s Ranksum test to assess whether the medians of the two populations are significantly different. All values with P < 0.05 were considered to be significantly different. All the computations were performed in MatLab.

## Results

Fig 1 shows the efficiency-minus-density curves for the EEG-based functional networks, which analysis has been proposed to study the economic structure of brain networks [7, 39, 45]. Our results showed that EEG-based brain functional networks are less efficient (in terms of global efficiency measure) than random networks. For all density values and frequency bands, random networks had higher global efficiency than brain networks (P < 0.05). Furthermore, the brain networks had the optimal economic wirings in higher density values (~ 0.2) as compared with the random ones (~ 0.12). In other words, EEG-based brain networks require more wirings than random ones for economic efficiency. Global efficiency is indeed measuring the general communicability of a network. Our results indicate that brain networks have not been evolved in a way to be optimal for communicability of its regions with each other, or at least, there are other mechanisms controlling the evolution of brain networks.

**Fig 2:**
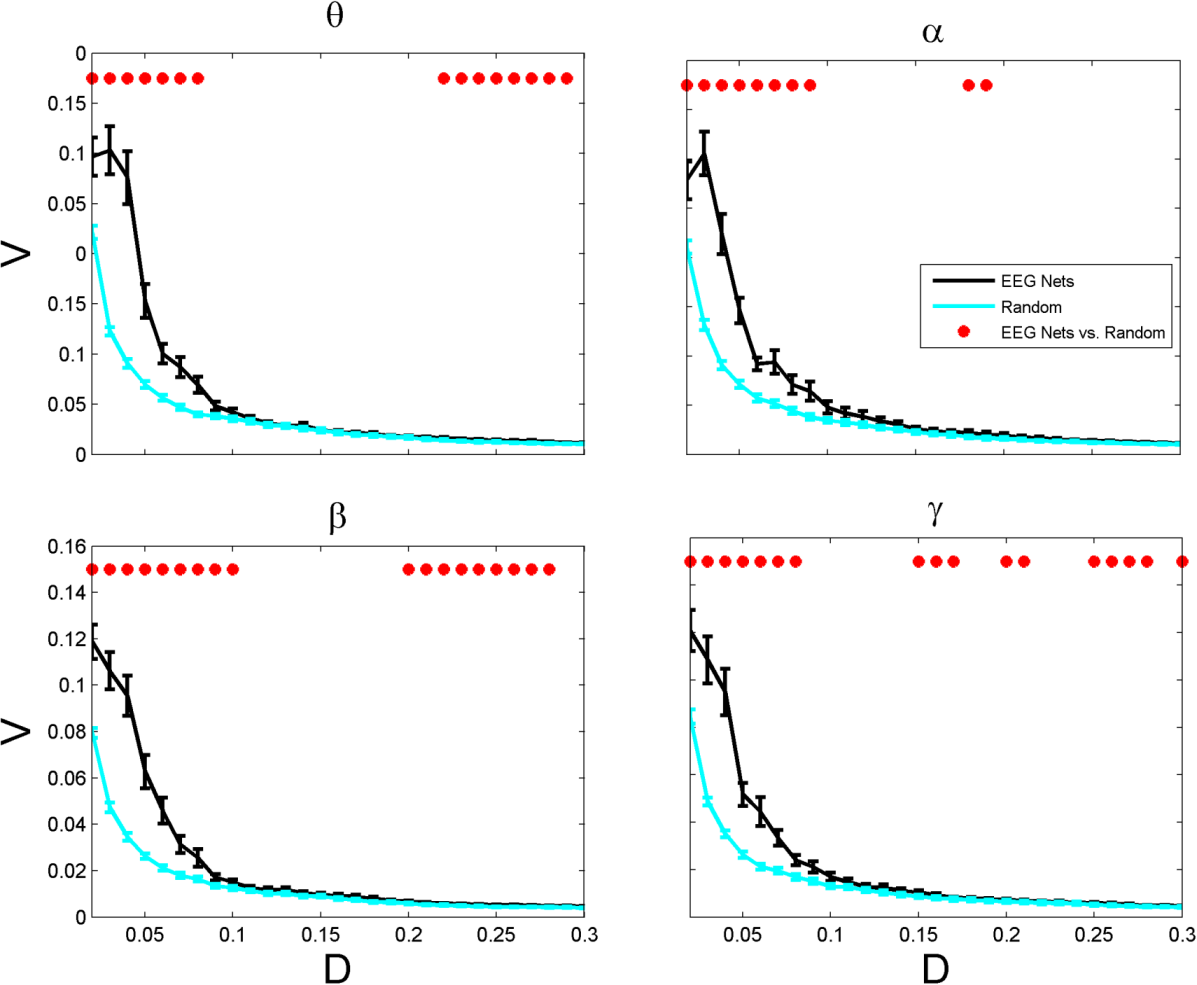
Vulnerability *V* (as defined in Eqs (3) and (4)) as a function of density in EEG-based brain functional networks and the corresponding randomized versions. Other designations are as Fig 1.

**Fig 3:**
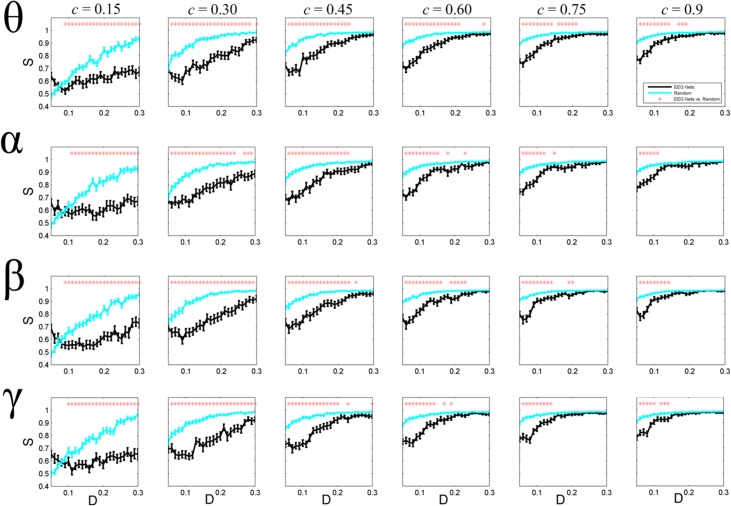
Cascaded failures in EEG-based brain functional networks and the corresponding randomized versions. The graphs show the normalized size of the survived nodes (the mean value with bars indicating the standard error) as a result of a cascaded failure. The graphs are for different values of parameter *c* (see Eq (6) for details), where higher values of *c* indicate higher load capacity for the nodes, and consequently, better resiliency against a cascaded failure. The failure starts by removing the node with the highest betweenness centrality value, then redistributing the loads, and then removing the nodes for which the load is higher than the capacity. The process is repeated until no further removal is needed and a steady state solution is obtained. Finally, the percentage of the survived nodes (*S*) is plotted as a function of the network density.

Figs 2 and 3 show to how much extent the brain networks are failure resilient as compared to random ones. For a broad range of density values, the brain networks have higher vulnerability (P < 0.05) than random networks (Fig 2). Vulnerability metric indicates how much vulnerable the network is, if its nodes are removed from the network. For example, vulnerability of 0.1 indicates that the network loses up to 10% of its efficiency, when a single most vulnerable node is removed from the network. Our results showed that sparse brain networks (with density values smaller than 0.1) were more vulnerable than random ones across all frequency bands (P < 0.05). Although high density networks in alpha band did not significantly differ in their vulnerability values, we identified a significant difference in theta, beta and gamma bands, for which brain networks had higher vulnerability than random ones.

Fig 3 shows the normalized size of the survived nodes (*S*) as a function of network density, when a cascaded failure happed in the network. Cascaded failures are devastated failures in which breaking down a component leads other components not to tolerate the extra load, and consequently fail. Studying how the brain reacts against cascaded failures is an important issue, since information processing in the brain is highly hierarchal [46], and the failure can rapidly propagate through the network. In all frequency bands, random networks showed significantly better resiliency against cascaded failures as compared to the brain networks (P < 0.05). For small values of *c*, the statistical significant is widespread across all density levels (*c* indicates the relation between the capacities of the nodes with their initial loads such that the higher the *c*, the higher the tolerance of the nodes; see Methods for detailed explanation). However, as *c* increased, we found no significant differences for high density values.

## Conclusion and Discussion

Graph theory tools have been extensively applied to study anatomical and functional networks of human brain [3, 4]. The brain is an extraordinary complex network, with nodes representing the individual regions and links the anatomical/functional connections between them, and studying its properties can help us better understand the brain. *Connectomics* view, which deals with comprehensive mapping the brain interconnections, gets increasing attention in understanding brain disorders [3, 47]. Graph theoretical analysis of anatomical/functional brain networks in health and disease has provided their specifications. Studying how removal of some components affects the network functionality is important for the brain networks, since some brain regions might, for some reasons, lose functionality and fail to communicate with other regions.

In this work we investigated failure tolerance of EEG-based brain functional networks and compared with that of properly randomized networks. Our results showed that global efficiency (analogous to the inverse of average path length) of brain networks were significantly less as compared to the corresponding random networks. This means that the wirings of brain functional networks have been placed primarily not for providing optimal communication between the regions, but for other neurophysiological reasons for which the regional connections are needed. Indeed, if global efficiency had a major role in optimizing the neuronal connections, the brain networks should have comparable efficiency with random ones. We also studied how removing the nodes affected the network properties. To this end, we performed two experiments. In the first experiment, the vulnerability of the networks was studied; brain networks were more vulnerable than random ones indicating that they significantly lose the normal functionality if the vulnerable regions are subject to attack. Vulnerability of the brain networks was 0.12 in some cases, which means that the network loses 12% of its efficiency when only a single node (out of 111 nodes) is removed from the network. As the second experiment, we studied how brain networks response to cascaded failures. The results revealed weaker resiliency of brain networks as compared to random ones against cascaded failures. We showed that by simulating the cascaded failure by first failing the highly loaded nodes, and then propagating it along the network, the brain networks had significantly less survived nodes than randomized versions.

In summary, our results suggest that brain networks are fragile to targeted attacks. However, due to short period of EEG recordings (3–4 minutes), the interpretations of the results are limited and should be replicated with longer EEGs. Also, to have better understanding of resilient behaviour of brain networks, further experiments should be carried out using other neuroimaging techniques such as fMRI, MEG and DTI.
